# Race-Discordant School Attendance and Cognitive Function in Later Life

**DOI:** 10.1093/geroni/igab046.2633

**Published:** 2021-12-17

**Authors:** Dawn Carr, John Reynolds

**Affiliations:** Florida State University, Tallahassee, Florida, United States

## Abstract

Early schooling plays an important role in shaping cognitive development, both due to the level of academic rigor and the social environment of primary and secondary schools. This is reflected in current racial disparities in cognitive function in later life. Older minorities who attended predominantly White schools with more resources experienced significant cognitive benefits. This study explores whether there are benefits to cognitive functioning in later life from having attended socially diverse schools in early life. We examine the effects of having attended schools composed primarily of different race peers—race discordant schools (RDS)—among Black, Hispanic, and White older adults. Using retrospective and prospective data from the Health and Retirement Study, we examine the association between RDS exposure and four measures of cognitive function (working memory, episodic memory, mental status, overall cognitive function). We assess function at age 55 and 70, and examine change in functioning between age 55 and 70. We find that RDS exposed Blacks and Hispanics experience significant benefits in cognitive function at age 55, but only Blacks experience benefits at age 70. RDS exposed Whites reported higher overall working memory at age 70 relative to Whites in non-RDS schools, suggesting a cognitive benefit from diversity. Results suggest that exposure to more racially diverse school environments have potentially beneficial effects on cognitive function over the life course. Our findings suggest that the cultivation of diversity in schools could be an important long-term public health investment.

